# In situ conservation of traditional vegetable diversity in Wa homegardens in southwestern Yunnan, China

**DOI:** 10.1186/s13002-021-00479-4

**Published:** 2021-09-08

**Authors:** Hua Shao, Rosemary Hill, Dayuan Xue, Jingbiao Yang

**Affiliations:** 1 College of Life and Environmental Sciences, Minzu University of China, Beijing, 100081 China; 2 Land and Water, Commonwealth Scientific and Industrial Research Organisation, Cairns, Queensland 4878 Australia; 3 Key Laboratory of Ecology and Environment in Minority Areas (Minzu University of China), National Ethnic Affairs Commission, Beijing, 100081 China

**Keywords:** Traditional vegetable diversity, In situ conservation, Homegardens, Traditional culture, Wa people

## Abstract

**Background:**

Homegardens are in situ conservation sources of germplasm diversity for overcoming homogenous germplasm problems in industrial agricultural systems. The Wa people constitute a long-dwelling ethnic group mainly in southwestern Yunnan with a unique culture and rich knowledge of traditional vegetables. We hypothesized that traditional vegetable varieties are well conserved in Wa homegardens because Wa culture promotes the preservation of traditional vegetables. We surveyed vegetable varieties and the practices that are involved in the conservation of traditional vegetables in Wa homegardens, which could form the basis for in situ conservation.

**Methods:**

The methods were used including questionnaires and semi-structured interviews. Sixty homegardens were surveyed through purposive sampling in 6 Wa villages. We documented ethnobotanical information about vegetables in homegardens. Plant species were identified according to the *Flora of China*. And thematic analyses were conducted for in-depth interviews to identify the conservation factors for traditional vegetables.

**Results:**

Fifty-two vegetable species belonging to 16 families and 41 genera were recorded from 60 Wa homegardens. Fifty-five traditional vegetable varieties and thirty-six hybrids were recorded. Among all the villages, 23 ± 6 (average ± SD) traditional vegetable varieties per homegarden and 9 ± 3 (average ± SD) introduced varieties per homegarden were recorded. Local seeds were stored in 78% of households, with an additional 9% of households’ seed supplies coming from neighbors and relatives; the other 13% of households purchased local seeds from markets. In 83% of families, the female head was mainly responsible for the decision-making concerning traditional vegetables in homegardens; in 10% of families, the male head was responsible for decision-making, and a small percentage (2%) was determined by elderly people. Five percent of families made decisions jointly between male and female household heads.

**Conclusions:**

This study demonstrated that rich traditional germplasm diversity is harbored in Wa homegardens because of the unique culture and traditional knowledge of Wa communities, which are practiced daily with homegrown food plants. Local vegetable seed conservation and sharing systems help maintain germplasm diversity in the Wa community homegardens. Wa homegardens constitute a practical solution for protecting traditional germplasm diversity and maintaining traditional lifestyles.

## Background

Traditional vegetables are cultivated varieties that have arisen through a long history of selection and cultivation in small farming systems such as homegardens [1]. As the global population continues to increase, high malnutrition rates, insufficient energy sources, and a lack of essential nutrients such as vitamins continue to be major concerns [2]. However, traditional vegetables, as valuable sources of food and nutritional security, are underutilized and underrepresented in the global conservation system for plant genetic resources [3–5]. With the ongoing expansion of the global market economy and modernization of agriculture, high-yielding hybrid vegetables have become increasingly popular and valued, and the diversity of traditional vegetables is threatened with extinction [6].

Homegardens have been recognized as sources of high vegetable germplasm diversity, which can be essential for overcoming food-security problems, such as the loss of food sources from rapidly spreading diseases associated with homogenous germplasms in industrial agricultural systems [7–10]. Studies have emphasized that homegardens have many attributes related to plant diversity, multiple functions, and other economic benefits for farmers [11, 12]. These homegardens provide a high diversity of cultivated plants for self-sufficiency and social values underpinning and contributing to cultural diversity [13–15]. Furthermore, homegardens are more sustainable and adapted to local demands because of the planting of traditional varieties and the use of traditional management practices [16–18]. Diversity in homegardens can be affected by the interactions among spatial, environmental, demographic, social, economic, and cultural factors to influence agricultural practices [19, 20]. Gender is another factor that influences ownership and crop diversity in homegardens, and women and older people traditionally take responsibility for homegardens [21, 22].

With a population of approximately 429,700 in China, the Wa people are one of 55 ethnic minorities according to the reports of the sixth national census [23]. Wa people inhabit mainly mountainous areas referred to as the Wa Mountains, which are located in Cangyuan Wa Autonomous County and Ximeng Wa Autonomous County in southwestern Yunnan. The Wa language is an offshoot of the Palaungic branch of the Austroasiatic language family and has no history of written words [24]. Wa villages are located on hillsides, and traditional houses are constructed from thatch, bamboo, and timber. Each house has a fire pit inside to cook food and keep the house warm. Their staple foods are rice, corn, and buckwheat. The traditional belief of the Wa people is animism, and they worship nature, their ancestors, and both animals and plants [25]. The Wa mainly survives on abundant plant resources in the mountains and forests near the villages; they practice swidden agriculture and are involved in hunting and gathering. Wa people have a long history of vegetable farming and foster rich homegardens management practices and knowledge [26].

In southwestern Yunnan Province, homegardens are recognized as small agroecosystems and are used as part of in situ conservation strategies consistent with household socioeconomic practices of other ethnic groups [27, 28]. The region has a diverse ethnic composition, including Wa, Lahu, Dai, and 31 other ethnic minorities. Based on the previously reported influence of socioeconomic conditions among Wa people, traditional vegetable resources are an important part of their dietary culture [29, 30]. However, Wa homegardens and their management practices for various vegetables have not been reported in the literature.

This study demonstrates the traditional vegetable diversity and factors by which Wa households conserve traditional vegetable resources in their homegardens in Wa villages. Given the importance of traditional vegetables as essential ingredients in the Wa diet, we hypothesized that Wa villagers prefer to plant traditional vegetables and maintain the practices in homegardens because of their traditional culture. We expected to find that Wa people prefer to grow traditional vegetables in their homegardens for self-consumption. Women may take more responsibility for the decision-making of homegardens given the labor distribution among Wa households. And dietary custom and traditional culture could be the main factors to motivate Wa people to plant traditional vegetables. We argue that Wa homegarden management is a promising approach for conserving traditional vegetable resources to maintain dietary diversity and self-sufficiency.

## Methods

### Research area

**Fig. 1:**
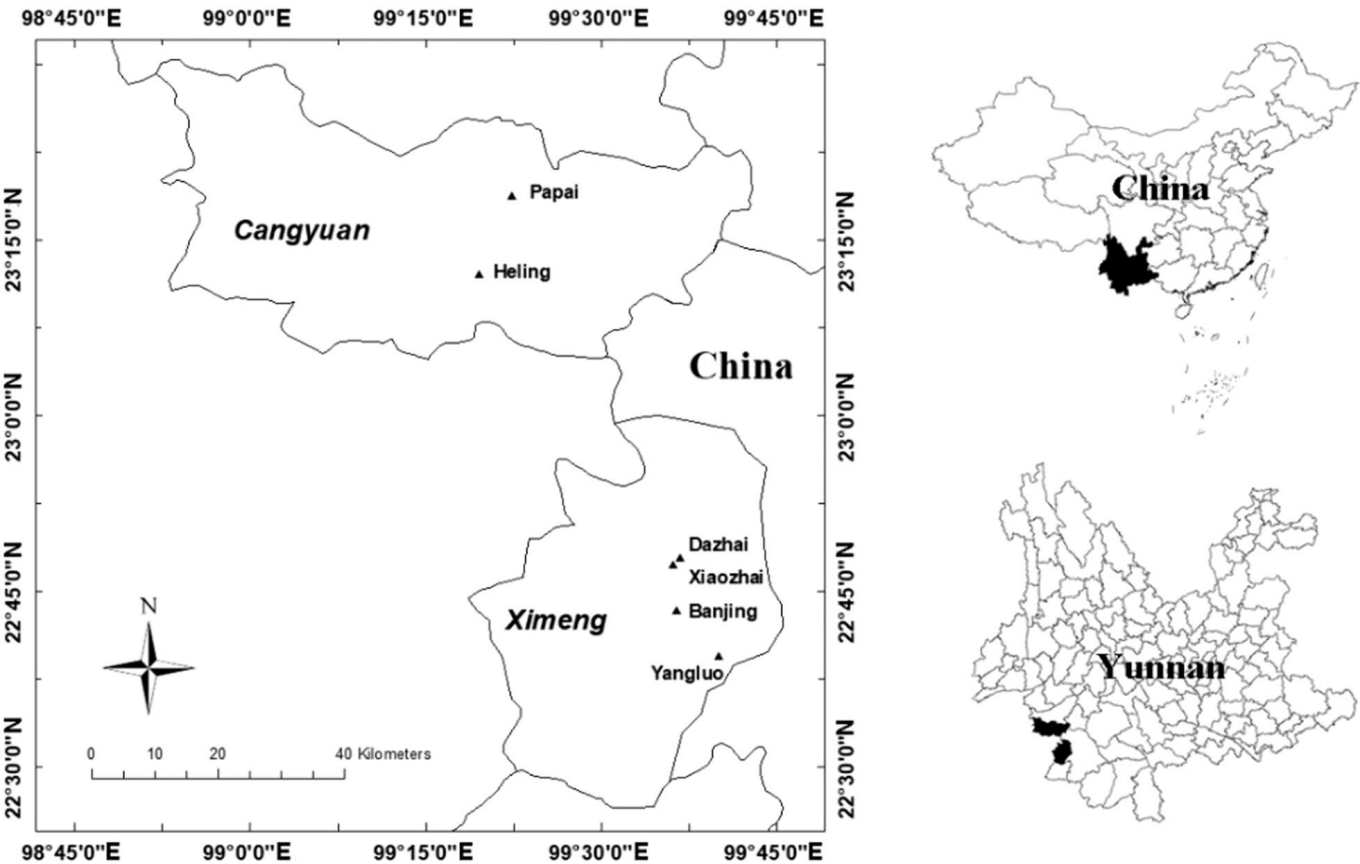
Geographic location of the study villages in Wa areas in China

The research area lies in the mountainous region of the southern part of the Nu Mountains at the border with Myanmar in the southwestern Yunnan province, China, between the western Lancang River and eastern Salween River (Fig. 1). This area, traditionally known as the Wa Mountains, straddles the Tropic of Cancer and has a subtropical climate with mild weather conditions. Affected by the warm and wet airflow from the Indian Ocean, rainfall is relatively abundant. The climate is also affected by the topography of this mountainous region.

This study was carried out in six villages of two administrative counties in May 2015, December 2015, May 2017, and December 2019. Cangyuan County is located between 98° 52′–99° 43′ E and 23° 04′–23° 30′ N. Cangyuan has a subtropical monsoon climate (mild and wet). The average amount of annual sunshine is 1862.5 h, the annual average temperature is 17.7 °C, and the annual average rainfall is 1747.2 mm [23]. Ximeng County is located between 99° 18′–99° 43′ E and 22° 25′–22° 57′ N. The region has a subtropical marine monsoonal climate affected by warm and humid conditions southwest of the Bay of Bengal. The annual average temperature is 15.3 °C, and the average amount of annual rainfall is 2758 mm, which is the highest in Yunnan Province. The highest elevation in this region is 2458.9 m above sea level, and the lowest elevation is 590 m. Due to this climate, the vegetation in these two counties is abundant and diverse, with a forest coverage rate of 37%.

Dazhai village, Xiaozhai village and Banjing village are in Zhongke town, Ximeng County. Yangluo village is in the town of Mengsuo in Ximeng County. Heling and Papai villages are in the town of Nuoliang in Cangyuan County (Table 1). Six representative villages were selected through discussions with elders, village heads, and local experts from the agricultural bureau in an initial survey visit [31], because these villages are inhabited mainly by Wa ethnic groups located far from the county center, and they are known for their long history and rich homegarden management experiences.

**Table 1:** Characteristics of six Wa villages

Village	Ximeng county				Cangyuan county	
	Zhongke town			Mengsuo town	Nouliang town	
	Dazhai	Xiaozhai	Banjing	Yangluo	Heling	Papai
Geographic location	99° 36′ E, 22° 47′ N	99° 36′ E, 22° 47′ N	99° 36′ E, 22° 43′ N	99° 40′ E, 22° 39′ N	99° 19′ E, 23° 12′ N	99° 22′ E, 23° 18′ N
Elevation (m)	918	862	802	1173	1694	1860
Distance from the county town/seat (km)	27.3	29.3	14.8	17.4	40	45
Number of households	136	138	83	570	62	270
Main industry	Crop farming, breeding	Crop farming	Crop farming	Crop farming	Crop farming	Crop farming

### Sampling and data collection

#### Sampling strategy for the homegarden survey

The household was the main sampling unit for the study. Sixty households were selected through a purposive sampling approach [32], following the advice of the village heads to identify the key informants. The survey was distributed to 10 households per village, accounting for at least 10% of the total households in each village. For reference, interviewees’ consent was based on household availability and interest.

A total of 60 questionnaires were distributed in the six villages, and 60 valid questionnaires were conducted to collect sociodemographic information on the participating farmers regarding age, gender, and education level [22] (Table 2). According to the age categorization, 46.67% of the informants were young people (21–40 years old), 45.00% were middle-aged (41–60 years old) and the remaining 8.33% were elderly (≥ 60 years old). A total of 86.67% of the informants were male. Regarding education level, 5.00% were uneducated, 51.67% attended primary school, 38.33% attended middle-high school, and 5% attended high school or beyond.

**Table 2:** Sociodemographic characteristics of the participants

Category	Gender		Age			Education level			
	Male	Female	21–40	41–59	≥ 60	Illiteracy	Primary school	Middle school	High school
Dazhai	10	0	5	3	2	0	4	6	0
Xiaozhai	6	4	6	4	0	1	7	2	0
Banjing	8	2	4	5	1	2	5	3	0
Yangluo	10	0	3	6	1	0	6	4	0
Heling	10	0	6	4	0	0	4	4	2
Papai	8	2	4	5	1	0	5	4	1
Quantity	52	8	28	27	5	3	31	23	3
Percentage (%)	86.67	13.33	46.67	45.00	8.33	5.00	51.67	38.33	5.00

#### Household survey of the management and seed system of homegardens

Key informant interviews were carried out by using both semi-structured and unstructured techniques. The key informants included household members, elders, and agro technicians. The interviews were held to gain an in-depth understanding of traditional practices associated with the management and seed system of homegardens. The first part of the interview aimed to determine the characteristics of homegardens, including their size and the cultivars grown. In the second part, seed management and sourcing, self-consumption through homegardens, and the gender of the person responsible for homegarden management were recorded. In the last part of the interview, threats and conservation issues were identified [33].

#### Identification of traditional vegetable cultivars

The criteria for traditional vegetables included the following: were considered heirloom crop vegetables, were local and culturally adopted, and were handed down from generation to generation [1]. With the local processes of selection and domestication, we consider that those plant species that were originally from other regions of the world and introduced centuries ago can be considered traditional species, including cultivated species and wild ones. Homegardens inventories included documentation of local names, edible parts, and eating methods of the vegetables planted. Focus group discussions (FGDs) were also held in each village to respond to an initial list of potential traditional vegetables and hybrid vegetable varieties planted in home gardens obtained from a literature review [30]. The nomenclature of all plants reported in our study followed that of *Flora of China* [34]*.* Vegetable varieties were jointly identified by local villagers, farmers, and agro technicians from the Agricultural Technology Extension Center. Ambiguous data in the reports were clarified via a FGD in each village. A final list of vegetable inventories was created, which could be further validated by farmers and local experts from the agricultural bureau. The homegarden system analysis and discussion that follow are based on the vegetable varieties identified.

### Data analysis

The data was analyzed to determine the taxonomic diversity of traditional vegetables planted in homegardens, the edible parts, the planting frequency of key vegetables, and the household characteristics of homes with gardens. The individual vegetable varieties in 60 homegardens were determined to identify the most important varieties in homegardens. In-depth interviews were analyzed by themes according to homegarden planting practices and factors. These factors included reasons why traditional vegetable resources have been conserved in homegardens; several themes reflecting social and cultural changes emerged through thematic analyses [35].

## Results

### Diversity and parts of traditional vegetables grown in Wa homegardens

**Fig. 2:**
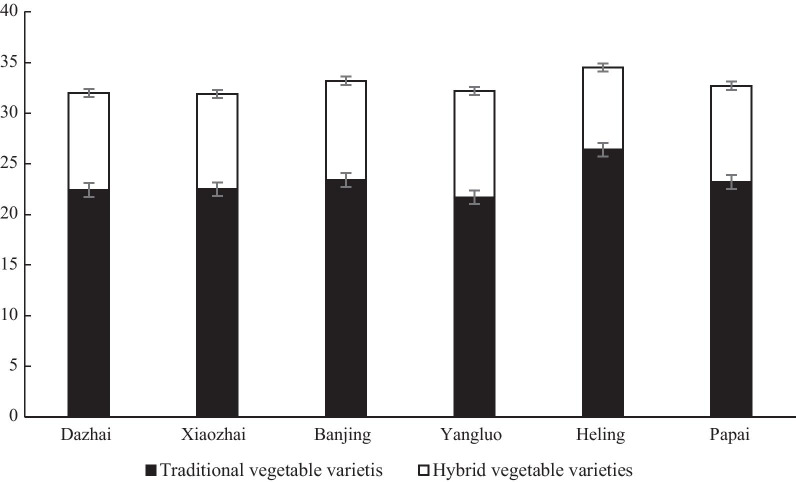
Portrait of vegetable diversity in six Wa villages

A total of 52 vegetable species belonging to 16 families and 41 genera were recorded in 60 Wa homegardens. Of the 91 vegetable varieties, 55 were local varieties, and 36 were hybrids, accounting for 60.44% and 39.56%, respectively, of all vegetable varieties grown (Table 3). Among all the villages, 23 ± 6 (average ± SD) traditional vegetable varieties per homegarden and 9 ± 3 (average ± SD) introduced vegetable varieties per homegarden were analyzed. There were more traditional varieties than hybrids in the six villages (Fig. 2). Out of the total traditional vegetable varieties, most were members of the Cucurbitaceae (10 varieties, 18.18%), followed by the Solanaceae (10 varieties, 18.18%), Brassicaceae (7 varieties, 12.73%), and Liliaceae (7 varieties, 12.73%) (Table 3).

**Table 3:** Traditional vegetables and their uses in Wa homegardens

Scientific name	Local name	Family	Edible parts	Eating method	Frequency of occurrence
*Allium chinense*	Jiao tou藠头	Liliaceae	Bulbs	Spice	23
*Allium fistulosum*	Pake da cong 帕科大葱	Liliaceae	Whole plants	Spice	7
*Allium fistulosum*	Xiao xiang cong小香葱	Liliaceae	Whole plants	Spice	60
*Allium sativum*	Bai pi suan白皮蒜	Liliaceae	Bulbs	Spice	32
*Allium sativum*	Yongguang da suan 永广大蒜	Liliaceae	Bulbs	Spice	3
*Allium hookeri*	Pie cai苤菜	Liliaceae	Roots, flowers	Spice	37
*Allium tuberosum*	Xi ye jiu cai细叶韭菜	Liliaceae	Leaves	Fry	60
*Amaranthus paniculatus*	Yi mi cai薏米菜	Amaranthaceae	Fresh leaves and stalks	Fry, Boil	60
*Benincasa hispida*	Lao mian dong gua 老缅冬瓜	Cucurbitaceae	Fruits	Fry	29
*Benincasa hispida*	Yuesong dong gua 岳宋冬瓜	Cucurbitaceae	Fruits	Fry	24
*Brassica chinensis*	Zi qing cai 紫青菜	Brassicaceae	Leaves, stalks	Fry, Boil	60
*Brassica chinensis*	Da qing cai 大青菜	Brassicaceae	Leaves, stalks	Fry, Boil, Pickling	29
*Brassica chinensis*	Wa qing cai 佤族青菜	Brassicaceae	Leaves, stalks	Pickling	54
*Brassica chinensis*	Yuan qing cai 圆青菜	Brassicaceae	Leaves	Pickling	49
*Brassica pekinensis*	Pake bai cai 帕科白菜	Brassicaceae	Leaves, stalks	Fry, Boil	28
*Brassica pekinensis*	Nangui bai cai 南归白菜	Brassicaceae	Leaves, stalks	Fry, Boil	17
*Capsicum annuum*	Chao tian jiao 朝天椒	Solanaceae	Fruits	Spice	38
*Capsicum annuum*	Xiaozhai la 小寨辣椒	Solanaceae	Fruits	Spice	13
*Capsicum annuum*	Talang la jiao 他朗辣椒	Solanaceae	Fruits	Spice	1
*Capsicum frutescens*	Xiao mi la 小米辣	Solanaceae	Fruits	Spice	60
*Capsicum frutescens*	Bai pi xiao mi la 白皮小米辣	Solanaceae	Fruits	Spice	46
*Capsicum frutescens*	Lao shu la jiao 老鼠辣椒	Solanaceae	Fruits	Spice	1
*Capsicum frutescens cv. Shuanlaense*	Shuan shuan la 涮涮辣	Solanaceae	Fruits	Spice	1
*Colocasia esculenta*	Zi yu 紫芋	Araceae	Corms	Fry, soup	3
*Colocasia esculenta*	Banshuai yu tou 班帅芋头	Araceae	Corms	Fry	23
*Colocasia esculenta*	Di shui yu 滴水芋	Araceae	Leaves, stalks	Soup, spice	54
*Colocasia esculenta*	Naka da ma yu 那卡大麻芋	Araceae	Corms	Soup	1
*Coriandrum sativum*	Xi ye yan sui 细叶芫荽	Apiaceae	Fresh leaves and stalks	Boil, salad, spice	51
*Cucumis sativus*	Di huang gua 地黄瓜	Cucurbitaceae	Fruits	Salad	57
*Cucurbita moschata*	Jin gua 金瓜	Cucurbitaceae	Fruits, fresh leaves, flowers	Fry	17
*Cucurbita moschata*	Lao mian nan gua 老缅南瓜	Cucurbitaceae	Fruits, fresh leaves, flowers	Fry	13
*Cucurbita moschata*	Xiao nan gua 小南瓜	Cucurbitaceae	Fruits, fresh leaves, flowers	Fry	33
*Dioscorea batatas*	Zi shan yao 紫山药	Dioscoreaceae	Tubers	Boil	2
*Dioscorea batatas*	Shan yao 山药	Dioscoreaceae	Tubers	Boil	39
*Dioscorea batatas*	Xi shan yao 细山药	Dioscoreaceae	Tubers	Boil	19
*Foeniculum vulgare*	Hui xiang 茴香	Apiaceae	Whole plants	Spice	2
*Lagenaria siceraria*	Hu lu 葫芦	Cucurbitaceae	Tender leaves	Fry	4
*Luffa cylindrica*	Si gua 丝瓜	Cucurbitaceae	Tender fruits	Fry	16
*Lycopersicon esculentum*	Xiao fan qie 小番茄	Solanaceae	Fruits	Salad	21
*Eryngium foetidum*	A Wa yan sui 阿佤芫荽	Apiaceae	Whole plants, fresh leaves, and stems	Spice	18
*Mentha haplocalyx*	Bo he 薄荷	Lamiaceae	Tender stem tips, leaves	Spice, Fry	27
*Momordica charantia*	Menge ku gua 勐阿苦瓜	Cucurbitaceae	Fruits	Fry, Salad	1
*Nepeta cataria*	Jing jie 荆芥	Lamiaceae	Fresh leaves	Spice	60
*Pachyrhizus erosus*	Hong shu 红薯	Leguminosae	Bulbs	Fry	3
*Perilla frutescens*	Bai su 白苏	Lamiaceae	Leaves, stalks	Spice	1
*Perilla frutescens*	Hei su 黑苏	Lamiaceae	Leaves, stalks	Spice, Salad	1
*Pisum sativum*	Lao zhai wan dou 老寨豌豆	Leguminosae	Seeds	Fry	14
*Pisum sativum*	Wangya wan dou 王雅豌豆	Leguminosae	Seeds	Fry	12
*Pisum sativum*	Hong wan dou 红豌豆	Leguminosae	Seeds	Fry	10
*Raphanus sativus*	Bai luo bo 白萝卜	Brassicaceae	Roots, leaves	Pickling	2
*Sechium edule*	Fo shou gua 佛手瓜	Cucurbitaceae	Tender stem tips, flowers, fruits	Fry	60
*Solanum melongena*	Bai qie 白茄	Solanaceae	Fruits	Fry	14
*Solanum melongena*	Zi qie 紫茄	Solanaceae	Fruits	Fry	40
*Vigna unguiculata*	Dou jiao 豆角	Leguminosae	Fresh pods	Fry	33
*Zingiber officinale*	Huang jiang 黄姜	Zingiberaceae	Rootstocks	Spice	54

The more frequently each traditional vegetable variety was planted by local Wa villagers in their homegardens, the more important and valuable it was in the Wa community. The most frequent vegetable varieties encountered in 60 homegardens included *Allium fistulosum*, *Allium tuberosum*, *Amaranthus paniculatus*, *Brassica chinensis*, *Capsicum frutescens*, *Nepeta cataria*, and *Sechium edule*. *Amaranthus paniculatus* is widely planted in Wa homegardens owing to its drought resistance, tolerance to infertile soil, high yield, and lack of diseases or insect pressure. *Allium fistulosum*, *Nepeta cataria*, *Allium tuberosum*, and *Capsicum frutescens* are used as spices in traditional foods, which indicates that members of Wa households select and plant local spicy vegetables in their homegardens for seasoning.

On the basis of the survey, we also found that, in addition to traditionally grown vegetables, 15 wild edible vegetables within 14 families were distributed in or around the yard (Table 4). These wild vegetables are largely used as spices to enhance the aroma and flavor of dishes and have other purposes, including medicinal and ornamental ones. These wild vegetables are readily accessible along the roadside, such as *Buddleja officinalis*, *Colocasia gigantea*, *Ensete wilsonii*, *Eryngium foetidum*, *Houttuynia cordata*, and *Mentha canadensis*. Some of the wild plants can be collected at the forest edge near the village, such as *Bombax ceiba*, *Buddleja officinalis, and Solanum americanum*.

**Table 4:** Wild vegetable plants in/near the homegardens in Wa villages

Scientific name	Family	Habitats	Other usages
*Acacia pennata*	Fabaceae	Forest	
*Aralia chinensis*	Araliaceae	Forest	Medicinal
*Bombax ceiba*	Malvaceae	Forest, forest edge, roadside	Ornamental, textile
*Buddleja officinalis*	Loganiaceae	Forest edge, roadside	Dyeing, medicinal
*Colocasia gigantea*	Araceae	Roadside	
*Ensete wilsonii*	Musaceae	Roadside	
*Eryngium foetidum*	Apiaceae	Roadside	Seasoning
*Houttuynia cordata*	Saururaceae	Roadside	Seasoning
*Mentha canadensis*	Lamiaceae	Roadside	Medicinal
*Oenanthe javanica*	Apiaceae	Low wetlands, shallow marshes, riverbanks	Medicinal
*Polygonum viscosum*	Polygonaceae	Roadside	Seasoning
*Portulaca oleracea*	Portulacaceae	Roadside	Medicinal
*Solanum americanum*	Solanaceae	Forest edge, roadside	Medicinal
*Taraxacum mongolicum*	Asteraceae	Roadside	Medicinal
*Zanthoxylum bungeanum*	Rutaceae	Forest, forest edge	Medicinal, seasoning

There are various ways to eat traditional vegetables, including frying them, boiling them, using them in salads, and using them for spices. These traditional eating methods essentially require Wa gardeners to grow an abundance of vegetables such that dietary needs are met. Among the 55 recorded traditional vegetable cultivars, their edible plant parts could be divided into nine categories: fruits, leaves, stalks, bulbs, flowers, seeds, roots, whole plants, and pods (Table 5). Fruits (20 species, 28.17%), mainly from plants in the Cucurbitaceae and Solanaceae families, are the most used plant parts for nutrition. For 19 species (26.76%), fresh leaves were the second most used part for eating by the Wa people. In households, the fresh leaves of traditional vegetables are eaten via multiple methods, including as seasonings, by frying, by boiling, and as salad ingredients. For five species (7.04%) of 55 cultivars, the flowers were consumed as ingredients of traditional foods. These findings showed that Wa villagers have an anthophagous (flower eating) culture.

**Table 5:** Numbers of species per edible plant part

Edible parts	Number of species	Percentage (%)
Fruits	20	28.17
Leaves	19	26.76
Stalks	10	14.08
Bulbs	7	9.86
Flowers	5	7.04
Seeds	3	4.23
Roots	3	4.23
Whole plants	3	4.23
Pods	1	1.41

### Functions of Wa homegardens that provide self-sufficient vegetables and occasional income for households

**Fig. 3:**
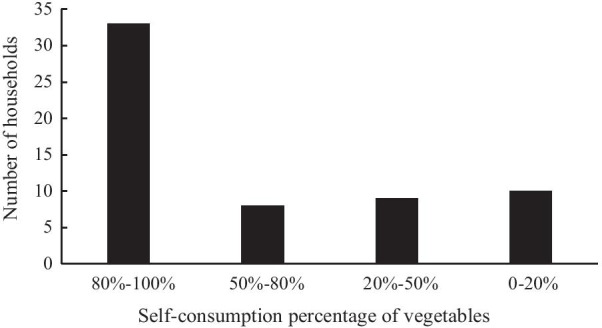
Percentage of total consumed vegetables from homegardens

The vegetables grown in the homegardens were used mostly for self-consumption and fulfilling owners’ needs. The number of households in which vegetables were grown in homegardens for self-consumption was relatively high (Fig. 3). Thirty-three out of 60 households accounting for 55% use 80%-100% of their vegetables. Thirteen percent of households have achieved self-sufficiency through the consumption of 50%-80% of the cultivated vegetables in their homegardens. Fifteen percent of households are self-sufficient through the consumption of 20%-50% of cultivated vegetables in their homegardens. Seventeen percent of households use 0–20% of the cultivated vegetables in their homegardens. Vegetables are used primarily for household consumption but are increasingly being used to generate cash incomes by several families. According to the interviewees, sometimes a surplus from consumption is sold to increase supplementary income for the families.

### Seed sourcing and management for vegetable restoration and conservation

#### Seed exchange networks in Wa communities

**Fig. 4:**
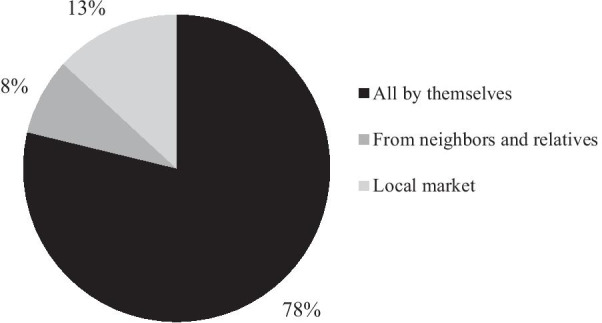
Proportion of households’ source of traditional vegetable seed supply

Seed storage and protection are essential components of traditional crop-related knowledge. Traditional seeding and breeding methods can promote the protection and inheritance of local vegetable germplasm resources. Among all the traditional vegetable seed sources, approximately 78% of the 60 household seed supplies depend on the maintenance and storage of the seeds by members of the household, and an additional 8% of household seed supplies come from neighbors and relatives (Fig. 4). When vegetable plants growing in homegardens have excellent characteristics such as color, quality, or resistance to insects, neighbors, and relatives can ask to exchange seeds. In this way, local people have access to a positive, regular, and reciprocal seed exchange system in local areas. Excellent local vegetable landraces are selected from generation to generation, which is conducive to the preservation and development of traditional vegetables.

Seed exchange practices occur not only within villages but also outside Wa communities. Exchanges between villages and towns occur in traditional markets—farmers sell local traditional vegetable seeds in markets. Approximately 13% of households purchase local seeds from these markets. Some farmers sell traditional vegetable seeds that have better quality and set the price themselves. Like in many of the local communities, seed exchange is not the main mechanism by which seeds are acquired in Wa communities, with most seeds coming from each households’ storage systems. In this context, it is not surprising that, although active, traditional seed exchange methods are fragmented and decentralized.

#### Seed storage practices

According to the interviews, households in Wa villages use primarily local storage methods to preserve the seeds of traditional vegetables planted in their homegardens for use the following year. Local seed storage practices are simple: the seeds are hung above the fireplace where meals are cooked daily in the households. This keeps the seeds directly in a dry and ventilated place to prevent mildew and predation by insects. The selection of seeds for saving is based on their color, resulting in food quality, resistance to environmental stresses, yield, and so on. Because of the simple breeding and selection methods of seeds, traditional vegetable landraces such as melons, beans, and peppers, which are easily harvested, are preserved better than are other vegetables.

Exchanges between villages and towns occur through traditional markets—farmers sell local traditional vegetable seeds for a long time in traditional markets. However, while the local families’ economic conditions have gradually improved, the management of traditional vegetable seeds has become increasingly threatened. Local farmers have started to stop preserving traditional seeds and instead have been chosen to go to seed stores to buy modern hybrid seeds. Unlike in villages’ traditional markets, seed stores now provide only modern hybrid seeds for farmers. One of the seed dealers said, “Farmers now prefer to buy modern seed because of the high production when they have enough money” (Interview, 16 December 2015).

#### Gender relations concerning homegarden management

**Fig. 5:**
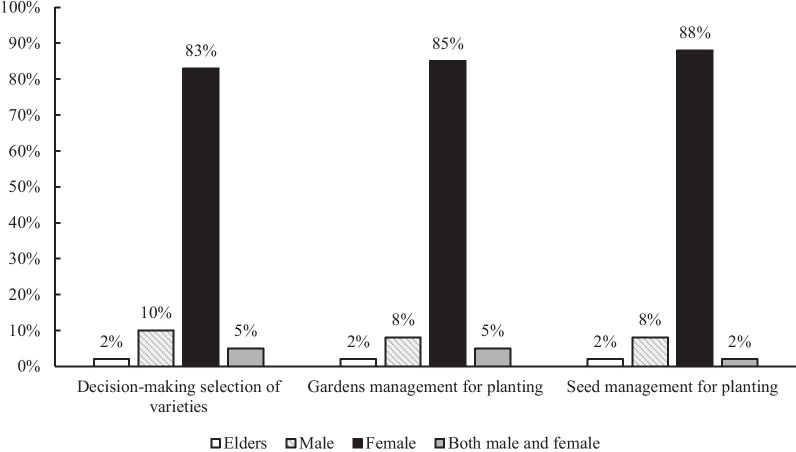
People responsible for preservation and cultivation of vegetables in homegardens (percentage/head of household)

Among the people we interviewed, most of the households had similar family structures and social relationships. With respect to homegarden management, 83% of families had a female head of household who made decisions about what kinds of vegetables to grow. Males accounted for 10% of the heads of households responsible for decision-making, and a small percentage (2%) of the decision-making was made by elderly people. Occasionally (in 5% of families), decisions were made by both male and female household heads. With respect to the management of homegardens, less than 8% of male heads of households make decisions. Similarly, regarding seed selection and breeding, female heads of households hold more of the responsibility (88%) (Fig. 5). The percentage of male household heads who are involved in garden management and are responsible for the selection of vegetable varieties and their preservation and cultivation is smaller than that of female household heads. However, male household heads are involved more in the decision-making concerning homegardens than in the actual management of those gardens.

#### Factors influencing the conservation of traditional vegetables in homegardens

**Fig. 6:**
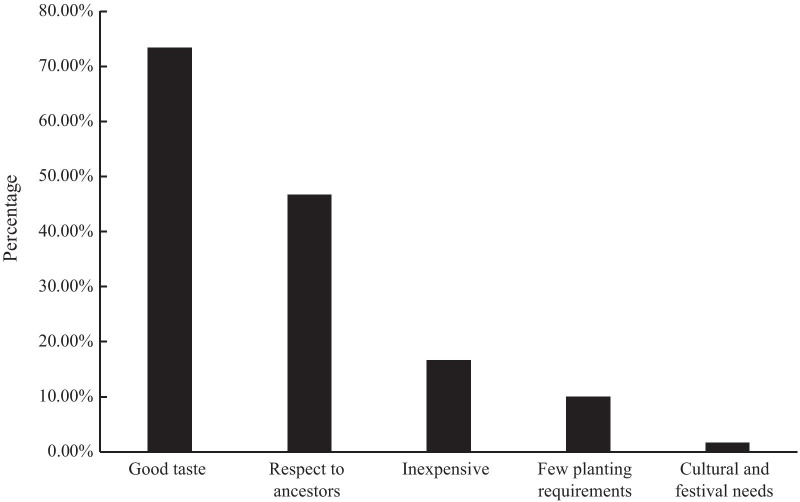
Factors for continuing to grow traditional vegetables according to Wa respondents

The participants noted five main factors for continued cultivation and selection of traditional vegetables in their homegardens: having good tastes (73.33%), honoring their ancestors by maintaining their traditions (46.67%), being inexpensive (16.67%), having few planting requirements (10.00%), and being needed for cultural purposes and festivals (1.67%) (Fig. 6).

Good taste is the fundamental reason Wa villagers keep planting traditional vegetables in their homegardens. According to Wa villagers, traditional vegetables typically have a more robust flavor than modern hybrid vegetables do, which is the main reason traditional vegetables are used in their daily meals. In addition, Wa farmers believed that many traditional vegetable varieties have adapted to local soil and climatic conditions through the millennia of cultivation and have superior traits or good palatability.

Nearly half of the respondents believed that maintaining traditional vegetables was critical to honor and respect their ancestors. Wa people worship and are conscious of their ancestors; Wa people value traditional vegetable varieties and grow them across generations to honor their ancestors.

In total, 16.67% and 10.00% of Wa villagers mentioned low planting costs as a requirement for not abandoning growing traditional vegetables in their homegardens. For example, *Allium fistulosum* has a strong, pungent, spicy taste; is highly resistant to disease, and is easily cultivated. These features help Wa villagers save on labor and financial resources needed to manage their gardens. Local people also prefer to grow traditional varieties from seed in their homegardens and without the use of fertilizers. Even though modern hybrid vegetables are more productive than traditional vegetables are, people would have to continually spend more money buying vegetable seeds and fertilizer from the market. Resource input and outputs are not proportional in such small systems; one of the farmers said the following:

“The traditional vegetables are easier to manage in homegardens. We do not need to spend too much time on pest control and fertilization with traditional vegetables, but as for modern vegetable varieties, they are easily threatened by pests and diseases, and we need to spend time spraying pesticides and applying chemical fertilizers to achieve high yields. It’s not always worth the effort.” (Interview, 3 August 2015).

A total of 1.67% of the respondents mentioned that culture and festivals make traditional vegetables vital to Wa customs. Although the traditional vegetables for these needs account for only a small proportion of all the vegetables grown, they have unique characteristics and should not be ignored. For instance, Wa people traditionally eat *Brassica chinensis* as a traditional dish during the New Year to bless the whole family for the coming year. Moreover, *Colocasia esculenta* (Di shui yu) and *Eryngium foetidum* (A Wa yan sui) are essential seasonings due to their particular flavor within the traditional chicken rice porridge Wa dish, and it is customary for Wa families to host guests and celebrate these festivals.

## Discussion

### Vegetable diversity and dietary culture

As revealed in other studies of homegardens in southwestern Yunnan Province [28, 36], the Wa community has a wide variety of choices and uses of vegetables from many families and different genera in their homegardens. Wa villagers have not limited themselves to specific families and genera in their cultivation and utilization of traditional vegetables but instead use a wide range of species, which reflects the diversity and universality of vegetable resource use in home gardens. Such diversity reflects both the rich germplasm biodiversity cultivated by members of Wa households and the complexity of traditional dietary habits in the Wa community.

Several factors are explaining why traditional vegetables are well maintained in Wa homegardens. First, the choice of vegetables to grow is based on cultural value, dietary culture, taste preferences, market accessibility, and household needs; these choices extend to wild plants that are brought into and maintained in homegardens. Wa people’s culinary culture involves mixing many vegetables and meats to enhance the taste and nutritive values of dishes, which fosters traditional knowledge of various eating methods for the different edible parts. Demand for traditional dishes also contributes to the preservation of several essential vegetable varieties. For example, Wa people prefer to eat the traditional Chicken Rice Porridge dish, which is made from fennel, tabasco pepper, mint, garlic, spring onion, cilantro, *Allium hookeri*, chicken, and rice*.* This dish is served when hosting guests and friends to show great respect, reflecting the cultural links with vegetables in Wa society. Additionally, the preference for spicy food customs has led to the maintenance of pepper varieties in the homegardens throughout history [26]. Second, the pursuit of health also promotes the diversity of vegetables used by Wa people. The daily Wa diet thus has several functions, including providing nutrients and health care knowledge [37]. Third, homegardens provide households with fresh, diverse vegetable supplies, contribute to self-sufficiency, and provide occasional income while maintaining in situ vegetable diversity. Moreover, homegardens not only maintain rich germplasm biodiversity but also enhance the inheritance of relevant ethnic and cultural practices as well as traditional knowledge [38, 39].

### Management of homegardens

Seed systems are an essential component of enhancing community resilience, as seed security has several direct links to food security [40, 41]. The circulation of seeds among farmers is central to agrobiodiversity conservation and dynamics [42]. A local seed supply enables local reproduction of seeds by farmers via local seed selection, production, and conditioning practices [43]. All of these practices depend on the ongoing transmission of traditional knowledge about seeds across generations. Local Wa people have a positive, regular, and reciprocal seed exchange system in local areas. Excellent local vegetable varieties are selected from generation to generation for preservation and the development of traditional vegetables. The seed exchange is not the primary mechanism for seed acquisition in Wa communities, with most seeds coming from each households’ own storage. In this context, it is not surprising that, although active, traditional seed exchange methods are fragmented and decentralized.

Homegardens are generally managed by one individual or a couple of family members, mainly female heads of households. In one study, women were aware of homegarden conservation for the conservation of agrobiodiversity in homegardens [44]. In the families of the present study, women were generally responsible for planting and managing vegetables, and for selecting and breeding, and their knowledge of traditional vegetable cultivation and preservation was well preserved among female groups. Women in the Wa family are responsible mainly for the cultivation of vegetables in the homegarden. These findings add weight to those of previous studies that identified the significant positive influence of women on the use, management, and conservation of biodiversity through their roles in seed selection, seed storage, and the use of wild plants for food and medicine [45, 46].

### Conservation threats for traditional vegetables

Although homegardens in Wa villages provide in situ conservation of traditional vegetables, rapid socioeconomic changes and the infiltration of foreign cultures are challenging the ongoing maintenance of traditional vegetables. As small agricultural systems, homegardens have always been neglected by policymakers. The economic demand for higher wages pulls farmers toward urban labor, and large-scale rural agricultural development has substantially reduced the rural labor force. Instead of being satisfied with the self-sufficient traditional agricultural production lifestyle, young people choose to go out to work or engage in other industries, which results in the gradual decline in traditional cultivation practices. Agricultural policies have led to the introduction or have partially subsidized hybrid vegetable varieties, and the government encourages local households to plant hybrids for increased yields. In the future, this will increase the homogeneity of vegetables planted in homegardens, which will ultimately consist primarily of modern hybrids. Either of these factors will result in the gradual disappearance of a large number of traditional vegetable varieties. A potential consequence is that local farmers, especially women, could subconsciously lose traditional knowledge of seed selection and breeding of traditional vegetables in their homegardens [47].

## Conclusions

This study suggests that rich, traditional vegetable diversity is maintained in homegardens by members of households in rural villages in Wa communities in southwestern China. In total, 52 plant species within 16 families and 41 genera were recorded as being present in Wa homegardens in the study area. A total of 91 vegetable varieties, including 55 local varieties and 36 hybrid varieties, were recorded. The continued planting and use of traditional vegetables from diverse seed sources in homegardens contribute to the conservation of germplasm diversity. Members of households with homegardens maintain and protect the diversity of traditional vegetables through their seed management practices, which involve both saving and exchanging seeds. The cultural preference for the traditional vegetables among the Wa people plays a positive role in the protection and utilization of traditional vegetable resources; vegetables that taste better have been continued to be cultivated in homegardens. Our results reinforce the evidence that maintaining homegardens can be practical in situ conservation solution for the protection of traditional resources. Policymakers should take homegardens into consideration in land planning for rural communities to maintain small-scale agricultural function and encourage farmers, especially women, to maintain homegardens for agrobiodiversity, in situ conservation, protection of traditional varieties, and maintenance of traditional knowledge held by ethnic people in the local communities.

## Data Availability

All the data generated or analyzed during this study are included in this published article.
